# Real-world comparison between neoadjuvant FLOT therapy and DCF therapy for resectable esophagogastric junction adenocarcinoma

**DOI:** 10.1007/s10388-025-01168-x

**Published:** 2025-11-07

**Authors:** Kazuhiro Shiraishi, Shun Yamamoto, Toshiharu Hirose, Hiroshi Imazeki, Kazuki Yokoyama, Yoshitaka Honma, Taiki Hashimoto, Tairo Kashihara, Daisuke Kurita, Koshiro Ishiyama, Junya Oguma, Hiroshi Igaki, Hiroyuki Daiko, Yasuyuki Seto, Ken Kato

**Affiliations:** 1 Department of Head and Neck, Esophageal Medical Oncology, National Cancer Center Hospital, 5-1-1 Tsukiji, Chuo-ku, Tokyo, 104-0045 Japan; 2 Department of Gastrointestinal Medical Oncology, National Cancer Center Hospital, Tokyo, Japan; 3 Department of Diagnostic Pathology, National Cancer Center Hospital, Tokyo, Japan; 4 Department of Radiation Oncology, National Cancer Center Hospital, Tokyo, Japan; 5 Department of Esophageal Surgery, National Cancer Center Hospital, Tokyo, Japan

**Keywords:** DCF, Esophagogastric adenocarcinoma, FLOT, Neoadjuvant chemotherapy

## Abstract

**Backgrounds:**

In Western countries, the standard perioperative treatment for resectable locally advanced esophagogastric junction adenocarcinoma (EGJ-AC) is 5-fluorouracil, oxaliplatin, and docetaxel (FLOT) therapy based on the results of the FLOT4 and ESOPEC trials. On the other hand, there was little evidence based on optimal perioperative treatment for resectable locally advanced EGJ-AC in Japan. Our previous report showed that neoadjuvant docetaxel, cisplatin, and 5-fluorouracil (DCF) therapy demonstrated modest efficacy for resectable locally advanced EGJ-AC. Therefore, we compared neoadjuvant DCF to FLOT therapy in terms of efficacy and safety in this study.

**Methods:**

We retrospectively analyzed the data of patients who received DCF or FLOT therapy for resectable EGJ-AC between 2015 and 2024 in our hospital. We assessed the R0 resection rate, histopathological response, disease-free survival (DFS), overall survival, and adverse events.

**Results:**

Thirty-two patients in the DCF therapy group and 20 patients in the FLOT therapy group were analyzed. The patients’ characteristics in the DCF group and FLOT group were as follows: median age, 63/59 years; ECOG PS 0, 66%/85%, respectively. The pCR rate was numerically higher in the FLOT group (20%) compared with the DCF group (3%) (p = 0.07). Similarly, the 1-year DFS rate was higher in the FLOT group (93%) than in the DCF group (68%) (p = 0.02), although this difference did not remain statistically significant after adjustment for baseline factors. Febrile neutropenia was significantly lower in the FLOT group (0%) than in the DCF group (12.5%).

**Conclusions:**

Neoadjuvant FLOT therapy is well-tolerated and has comparable short-term efficacy to DCF therapy.

## Introduction

Esophagogastric junction adenocarcinoma (EGJ-AC) shares clinical characteristics with esophageal adenocarcinoma (EAC) and is increasingly diagnosed in Japan [1–8]. While resection is the primary curative approach for resectable EGJ-AC, surgery alone is associated with poor outcomes.

In Western countries, standard treatment has evolved from surgery alone to perioperative therapy. The CROSS trial demonstrated the benefit of neoadjuvant chemoradiotherapy (NACRT) with a median OS of 43.2 months and 3-/10-year survival rates of 54% and 36% [11]. More recently, FLOT (5-FU, oxaliplatin, docetaxel) has become the standard based on the FLOT4 trial, which demonstrated superior overall survival with perioperative FLOT compared to ECX/ECF (50 vs. 35 months) [12], and the ESOPEC trial, which showed improved overall survival with perioperative FLOT compared to preoperative chemoradiotherapy (NACRT) (66 vs. 37 months) [13]. In Asia, adjuvant chemotherapy after surgery is more common, based on phase III trials including ACTS-GC [14], CLASSIC [15], and JACCRO-GC-07 [16], though EGJ-AC comprised less than 5% of patients. Recent trials have supported neoadjuvant strategies: PRODIGY (South Korea, including 7% of patients with EGJ-AC) showed OS benefit with DOS [17], RESOLVE (China, including 36% of patients with EGJ-AC) demonstrated improved OS with perioperative SOX [18], and DOS therapy showed a 24% pCR in Japan [19]. The ongoing JCOG2203 trial is evaluating NAC with DOS or FLOT in Japan [20].

DCF (docetaxel, cisplatin, 5-FU) therapy has shown promising efficacy in EGJ-AC, with a phase II trial reporting 100% R0 resection and 3-year OS of 60% [21]. A follow-up study reported 5-year OS of 53% and low local recurrence (4%) [22]. In ESCC, JCOG1109 demonstrated OS benefit of DCF over CF, and no superiority of NACRT over CF [23]. Thus, triplet neoadjuvant chemotherapy is a promising strategy in both ESCC and EGJ-AC, and DCF remains feasible with proper toxicity management.

Despite these developments, no direct comparison between FLOT and DCF has been reported. This study aims to evaluate the efficacy and safety of neoadjuvant FLOT versus DCF therapy followed by esophagectomy and mediastinal lymphadenectomy for resectable EGJ-AC.

## Materials and methods

### Patients

Between September 2015 and February 2024, patients were considered eligible for this study based on the following criteria: histologically confirmed EGJ-AC, clinical stage T1, N1-3, M0-1 or T2-3, N0-3, M0-1 (Union for International Cancer Control tumor-node-metastasis classification in 8th edition. The M1 disease in these three patients was limited to supraclavicular lymph node metastasis only.), Eastern Cooperative Oncology Group (ECOG) Performance Status (PS) 0–2. No previous treatment (chemotherapy or radiotherapy) for EGJ-AC. This study was conducted at a single institution, and clinical data were analyzed. This retrospective study complied with the regulations of the principles of Helsinki. This study was approved by the Institutional Review Board of the National Cancer Center Hospital (Approval No. 2020–287).

### The regimen of DCF and FLOT therapy

Neoadjuvant DCF consisted of DTX 70 mg/m^2^ and CDDP 70 mg/m^2^ on day 1, and 5-FU 750 mg/m^2^ on days 1–5, every 3 weeks, up to three cycles.

FLOT therapy consisted of 5-FU 2600 mg/m^2^, leucovorin 200 mg/m^2^, L-OHP 85 mg/m^2^, and DTX 50 mg/m^2^ on day 1, administered every 2 weeks for up to four cycles. After completion of chemotherapy, esophagectomy with D2 or D3 lymph node dissection was performed. The patient’s archived non-pathological complete response (pCR) was considered for adjuvant chemotherapy. One of the treatment regimens was to be treated with a triweekly combination regimen comprising oral S-1 (2-week administration followed by 1 week of rest, at a dose of 120 mg/day for patients with body surface area ≥ 1.5 m^2^, 100 mg for patients with body surface area of 1.25–1.5 m^2^, and 80 mg for patients with body surface area ≤ 1.25 m^2^) with intravenous DTX at 40 mg/m^2^ on day 1. Six cycles of this combination were to be delivered after one cycle of S-1 monotherapy (2-week administration followed by 1 week of rest), followed by further S-1 monotherapy (4-week administration followed by 2 weeks of rest) for 12 months.

### Assessments and statistical criteria

Efficacy was assessed based on treatment completion rates, histopathological response, disease-free survival (DFS), and OS. The treatment completion rate was defined as the proportion of patients who completed either ≥ 2 cycles of DCF therapy or ≥ 3 cycles of FLOT therapy. The histopathological response was evaluated according to the Japanese Classification of Gastric Carcinoma, 15th edition. DFS was defined as the interval between initiation of treatment and disease progression, incomplete (R1/R2) resection, relapse, secondary cancer, or death from any cause, whichever came first or was censored at the last date of confirmed survival without disease progression. OS was defined as the interval between initiation of treatment and death from any cause or censoring at the last date of confirmed survival.

Clinical staging was determined according to the 8th edition of the Union for International Cancer Control TNM classification by a multidisciplinary team, following a discussion based on endoscopy, thin-slice computed tomography, and positron emission tomography findings, as needed. Adverse events (AEs) were evaluated according to the Common Terminology Criteria for Adverse Events version 5.0, a Japanese translation of the JCOG version.

Categorical differences were evaluated using Fisher’s exact test for categorical data. A Student’s t test for continuous data was used to compare the characteristics of the two groups. Univariate and multivariate analyses were performed using Cox regression analysis to examine factors influencing DFS. Statistical tests were two-sided, and a p value < 0.05 was considered statistically significant. OS and DFS were estimated using the Kaplan–Meier method. When imbalances in baseline characteristics were observed between the two treatment groups, propensity score matching (PSM) was performed to minimize selection bias. The propensity score was estimated using a logistic regression model including covariates with significant or clinically relevant imbalance between the groups. One-to-one nearest-neighbor matching without replacement was performed with a caliper of 0.2. All data were analyzed using EZR version 4.2.2. (The R Foundation for Statistical Computing, Vienna, Austria).

## Results

### Patient characteristics

**Fig. 1:**
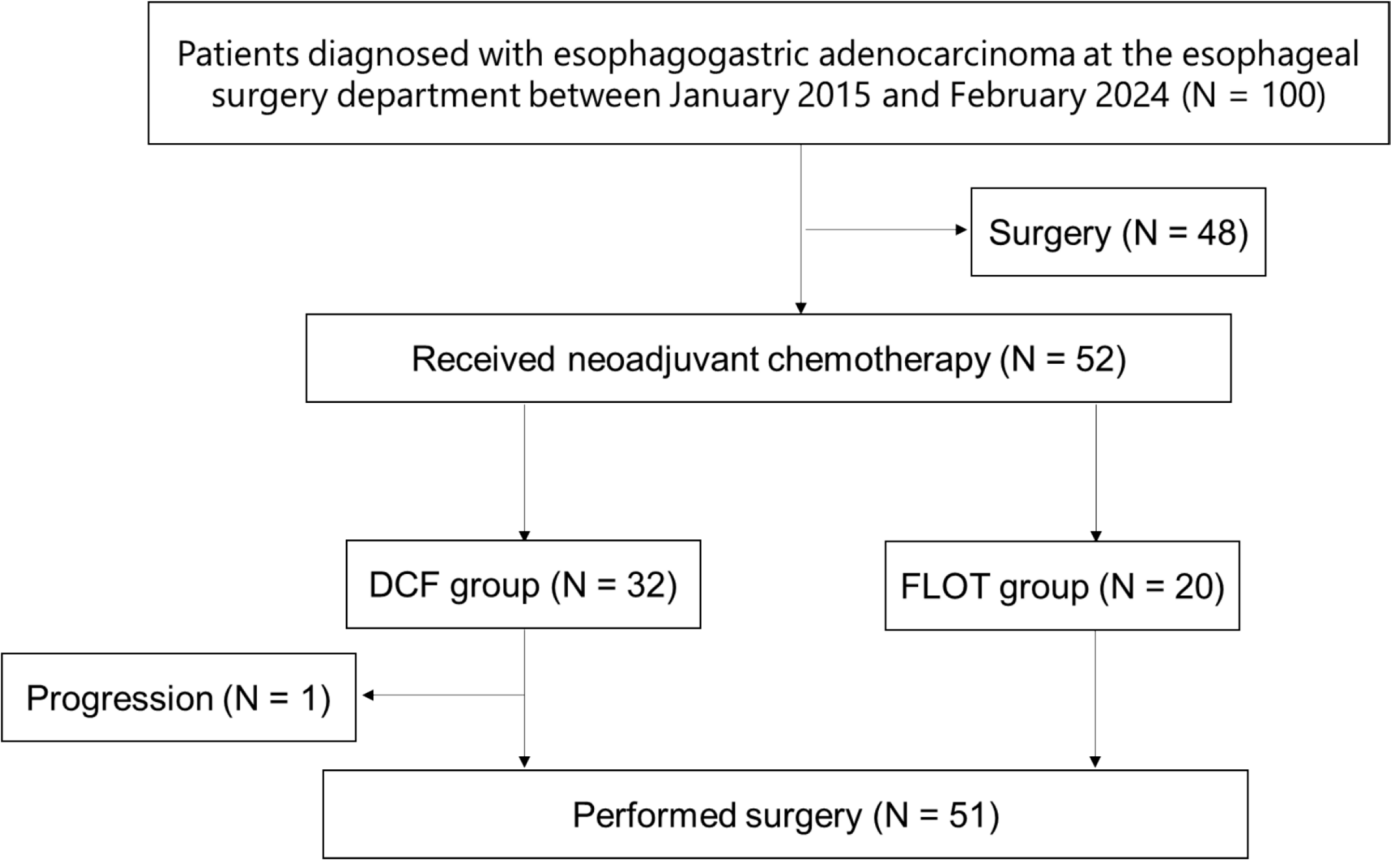
Patient flow diagram

From September 2015 to February 2024, 52 patients were enrolled in this study at a single institution; 32 received the DCF therapy group (DCF group), and 20 received the FLOT therapy group (FLOT group) (Fig. 1). The median age was 63 years (range 42–80) in the DCF group and 59 years (range 40–88) in the FLOT group, respectively. Patients’ characteristics are shown in Table 1A. There were no statistical differences in patients’ characteristics between the DCF and FLOT group except for the Siewert classification (*p* = 0.04).

**Table 1:** Patient characteristics before (**A**) and after (**B**) matched pair analysis

	DCF group n = 32 (%)	FLOT group n = 20 (%)	*p* value
(A) Before matched pair analysis			
Age, median (range), years	63 (42–80)	59 (40–88)	0.70
Sex, n			0.29
Male	31 (96.9)	17 (85.0)	
Female	1 (3.1)	3 (15.0)	
ECOG performance status, n			0.2
0	21 (65.6)	17 (85.0)	
1 ≤	11 (34.4)	3 (15.0)	
Siewert type, n			0.04
1	9 (28.1)	1 (5.0)	
2	21 (65.6)	19 (95.0)	
3	2 (6.0)	0	
T, n			0.08
T1b	1 (3.1)	0	
T2	3 (9.4)	7 (35.0)	
T3	28 (87.5)	12 (60.0)	
T4a	0	1 (5.0)	
N, n			0.21
N0	4 (12.5)	5 (25.0)	
N1	13 (40.6)	10 (50.0)	
N2	13 (40.6)	3 (15.0)	
N3	2 (6.0)	2 (10.0)	
M, n			0.28
M0	29 (90.6)	20 (100.0)	
M1	3 (9.4)	0	
Clinical stage, n			0.13
IIA	1 (3.1)	0	
IIB	0	1(5.0)	
III	14 (43.8)	14 (70.0)	
IVA	14 (43.8)	5 (25.0)	
IVB	3 (9.4)	0	

	DCF group n = 14 (%)	FLOT group n = 14 (%)	*p* value
(B) After matched pair analysis			
Age, median (range), years	59 (50–68)	59 (46–70)	0.83
Sex, n			0.48
Male	13 (92.9)	12 (85.7)	
Female	1 (7.1)	2 (14.3)	
ECOG performance status, n			0.21
0	11 (78.6)	12 (85.7)	
1 ≤	3 (21.4)	2 (14.3)	
Siewert type, n			1.00
1	1 (7.1)	1 (7.1)	
2	13 (92.9)	13 (92.9)	
3	0	0	
T, n			1.00
T1b	0	0	
T2	1 (7.1)	2 (14.3)	
T3	13 (92.9)	12 (85.7)	
T4a	0	0	
N, n			1.00
N0	2 (14.3)	3 (21.4)	
N1	7 (50.0)	6 (42.9)	
N2	4 (28.6)	3 (21.4)	
N3	1 (7.1)	2 (14.3)	
M, n			0.23
M0	13 (92.9)	14 (100.0)	
M1	1 (7.1)	0	
Clinical stage, n			1.00
IIA	0	0	
IIB	0	0	
III	9 (64.3)	9 (64.3)	
IVA	5 (35.7)	5 (35.7)	
IVB	0	0	

*ECOG* Eastern Cooperative Oncology Group

In the DCF group, 8 of the 32 patients terminated neoadjuvant therapy in the second cycle because of toxicities (grade 3 anorexia, n = 2; grade 4 neutropenia, n = 1), patient refusal (n = 3), or progression of disease (n = 2). Finally, 24 of the 32 patients (75%) received 3 cycles of DCF therapy. One patient revealed bone metastases before surgery and received systemic palliative chemotherapy. Thirty-one of the 32 patients (94%) underwent surgery. Twenty of 32 patients (63%) required at least one cycle of dose reduction of any drug. The mean relative dose intensity (actual dose/planned dose) was 0.87 for DTX, 0.84 for CDDP, and 0.86 for 5-FU, respectively. In the FLOT group, 3 of the 20 patients terminated neoadjuvant therapy in the second cycle, and 2 of the 20 patients terminated neoadjuvant therapy in the third cycle because of toxicities (grade3 anorexia, n = 2; grade2 allergy, n = 1), gastric ulcer due to tumor shrinkage (n = 1), patient refusal to continue (n = 1). Finally, 15 of the 20 patients (75%) received 4 cycles of FLOT therapy. All patients underwent surgery. Three of 20 patients (15%) required at least one cycle of dose modification of any drug, and 2 of 20 patients (10%) required treatment delay. The mean relative dose intensity (actual dose/planned dose) was 0.87 for DTX, 0.85 for L-OHP, and 0.87 for 5-FU, respectively. In the DCF group, no patients received adjuvant chemotherapy, and 4 patients received adjuvant chemotherapy in the FLOT group (p = 0.006), respectively. Treatment disposition is shown in Table 2.

**Table 2:** Treatment disposition

	DCF group n = 32 (%)	FLOT group n = 20 (%)	*p* value
Number of treatment cycles (median)	3 (1–3)	4 (2–4)	–
Treatment discontinuation due to AEs, n	3 (9.4)	5 (25.0)	0.24
With neither delay nor dose reduction	12 (37.5)	10 (50.0)	0.61
Treatment completion rate, n	32 (100.0)	17 (85.0)	0.05
Adjuvant chemotherapy			0.006
Yes	0	4 (20.0)	
Docetaxel + S-1	–	4 (20.0)	
No	32 (100.0)	16 (80.0)	

### Efficacy

Complete (R0) resection was achieved in 28 of 32 patients (88%), and R1 resection and R2 resection was in 2 each in the DCF group, and R0 resection was achieved in 19 of 20 patients (95%), and R1 resection was in 1 in the FLOT group. Thirteen patients (42%) in the DCF group and 15 patients (75%) in the FLOT group achieved a histopathological response of grade 1b or better (*p* = 0.02). The pCR was observed in 1 patient (3%) in the DCF group and 4 patients (20%) in the FLOT group, respectively (*p* = 0.07) (Table 3). In the two groups, significant imbalances were observed in tumor location according to the Siewert classification and clinical stage between the two groups. Therefore, PSM was performed based on these variables to adjust for background differences. After matching, 14 patients were included in each group, and baseline characteristics were well balanced (Table 1B). Still, the odds ratio (OR) for pCR was 0.25 (95% CI 0.03–2.24, p = 0.22), showing no statistically significant difference between the two groups.

**Table 3:** Univariate and multivariate analysis of DFS

Factors	Reference	N	Univariate analysis for DFS		Multivariate analysis for DFS	
			HR (95% CI)	*p value*	HR (95% CI)	*p value*
Regimen	FLOT	20	4.94 (1.13–21.5)	0.03	3.65 (0.76–17.4)	0.10
	DCF	32				
Siewert classification	Type1	10	–	–	–	–
	Type2	40	0.71 (0.25–1.99)	0.52	0.99 (0.33–2.95)	0.98
	Type3	2	8.23 (1.37–49.3)	0.02	5.72 (0.88–36.9)	0.07
Clinical T	1b, 2	11	6.86 (0.92–51.2)	0.06	5.10 (0.66–39.3)	0.12
	3, 4a	41				
Clinical N	0	9	1.73 (0.51–5.89)	0.38	0.83 (0.21–3.34)	0.80
	1, 2, 3	43				
Clinical stage	IIA, IIB, III	30	2.12 (0.89–5.05)	1.09	1.29 (0.46–3.61)	0.63
	IVA, IVB	22				

**Fig. 2:**
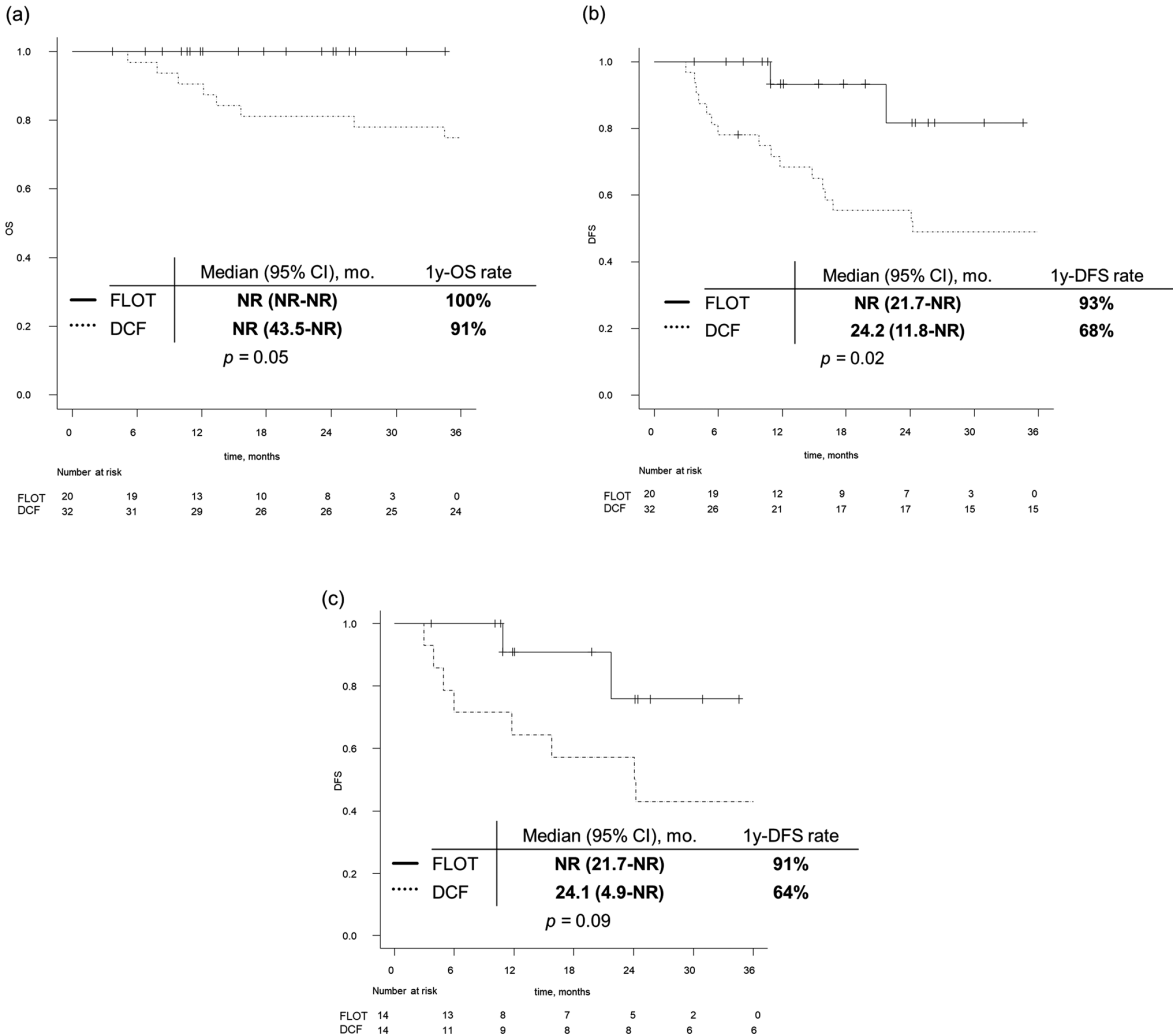
Kaplan–Meier analysis. **a** Overall survival of DCF group and FLOT group. **b** Disease-free survival of DCF group and FLOT group before matched-pair analysis. **c** Disease-free survival of DCF group and FLOT group after matched-pair analysis. *CI* confidence interval; *mo* months; *NR* not reached

Median follow-up time was 51.6 months (95% CI 24.2–64.4 months) in the DCF group and 16.6 months (95% CI 10.7–24.5 months) in the FLOT group, respectively. Median OS was NR in both groups. The one-year OS rate was 91% in the DCF group and 100% in the FLOT group (*p* = 0.05) (Fig. 2a). Median DFS was 24.2 months (95% CI 11.8–NR months) in the DCF group and NR (95% CI 21.7–NR months) in the FLOT group, respectively. 1-year DFS rate was 68% in the DCF group and 93% in the FLOT group (*p* = 0.02) (Fig. 2b). Cox regression analysis was performed to evaluate the potential impact of clinical factors (regimen, Siewert classification, clinical T stage, clinical N stage, and clinical stage) on DFS. In both univariate and multivariate analyses, none of these factors were found to be statistically significant (Table 3). After PSM was performed, the median DFS was 24.1 (95% CI 4.9–NR months) in the DCF therapy and NR (95% CI 21.7–NR months) in the FLOT therapy, respectively (*p* = 0.09) (Fig. 2c).

### Safety

Postoperative complications are shown in Table 4. In the DCF group, recurrent nerve palsy was the most common complication, occurring in 8 of the 31 patients (26%), followed by an anastomotic leak in 7 patients (23%). In the FLOT group, anastomotic leakage and pneumonitis were the most common complications in 2 of the 20 patients (10%).

**Table 4:** Surgical/pathological results

	DCF group n = 31 (%)	FLOT group n = 20 (%)	*p* value
Archived margin-free resection, n	28 (87.5)	19 (95.0)	1.00
Surgical procedure			0.38
RETML-4	9 (29.0)	10 (50.0)	
TLE	19 (61.3)	9 (45.0)	
MATHE	3 (9.7)	1 (5.0)	
Type of lymphadenectomy, n			0.001
1-Field	0	1 (5.0)	
2-Field	3 (9.7)	10 (50.0)	
3-Field	28 (90.3)	9 (45.0)	
The histological assessment of therapeutic response, n			0.04
1a	18 (58.1)	5 (25.0)	
1b	8 (25.8)	4 (20.0)	
2a	3 (9.7)	4 (20.0)	
2b	1 (3.2)	3 (15.0)	
3	1 (3.2)	4 (20.0)	
Pathological T stage, n			0.11
≤ ypT1	6 (19.4)	10 (50.0)	
ypT2	6 (19.4)	3 (15).0)	
ypT3	18 (58.1)	7 (35.0)	
ypT4	1 (3.2)	0	
Pathological N stage, n			0.20
ypN0	8 (25.8)	10 (50.0)	
ypN1	10 (32.3)	7 (35.0)	
ypN2	7 (22.6)	2 (10.0)	
ypN3	6 (19.4)	1 (5.0)	
Pathological complete response rate	1 (3)	4 (20)	0.07
Postoperative complications			0.92
Recurrent nerve palsy	8 (25.8)	1 (5.0)	
Anastomotic leakage	7 (21.9)	2 (10.0)	
Pneumonia	5 (15.6)	2 (10.0)	
Acute circulatory failure	3 (9.7)	0	
Lymphorrhea	1 (3.1)	0	
Postoperative mortality	0	0	

*RETML-4* Robotic esophagectomy with total mediastinal lymphadenectomy using four robotic arms, *TLE* Thoraco-laparoscopic esophagectomy, *MATHE* Mediastinoscopy-assisted transhiatal esophagectomy

The toxicity profiles of the DCF and FLOT groups are shown in Table 5. In the DCF group, hematological toxicity was the most common AE. Fourteen patients (43%) had grade ≥ 3 neutropenia. Febrile neutropenia (FN) was observed in 4 patients (12%). Anorexia and nausea were the most common non-hematological AEs, occurring in 27 patients (84%) and 28 patients (88%), respectively, and 3 (9%) patients experienced grade ≥ 3 anorexia and nausea, respectively. In the FLOT group, hematological toxicity was the most common AE. Twelve patients (60%) had grade ≥ 3 neutropenia, but no one experienced FN. Fatigue and anorexia were the most common non-hematological AEs, occurring in 10 patients (50%) and 8 patients (40%), respectively, and 1 (5%) patient of each among those who had grade 3. There were no treatment-related deaths in either of the neoadjuvant treatments.

**Table 5:** Adverse events

	DCF group n = 32 (%)		FLOT group n = 20 (%)		*p* value	
	Any grade	Grade. 3 ≤	Any grade	Grade. 3 ≤	Any grade	Grade. 3 ≤
Any events	32 (100.0)	19 (59.4)	20 (100.0)	15 (75.0)	1.00	0.37
Neutropenia	24 (75.0)	14 (43.8)	17 (85.0)	12 (60.0)	0.50	0.39
Anemia	29 (90.6)	0	17 (85.0)	0	0.66	1.00
Thrombocytopenia	14 (43.8)	0	9 (45.0)	0	1.00	1.00
Diarrhea	14 (43.8)	2 (6.3)	3 (15.0)	0	0.04	0.52
Nausea	28 (87.5)	3 (9.4)	3 (15.0)	0	< 0.01	0.28
Fatigue	13 (40.6)	0	10 (50.0)	1 (5.0)	0.57	0.39
Anorexia	27 (84.4)	3 (9.4)	8 (40.0)	1 (5.0)	< 0.01	1.00
Stomatitis or mucositis	16 (50.0)	2 (6.3)	2 (10.0)	1 (5.0)	< 0.01	1.00
Serum creatinine increased	12 (37.5)	0	1 (5.0)	0	< 0.01	1.00
Febrile neutropenia	4 (12.5)	4 (12.5)	0	0	0.15	0.15
Peripheral neuropathy	0	0	5 (25.0)	0	< 0.01	1.00
Allergy	0	0	1 (5.0)	0	0.39	1.00

## Discussion

In this retrospective study, although the relatively short follow-up period warrants cautious interpretation, in the overall analysis, the pCR rate tended to be higher and DFS significantly longer in the FLOT group compared with the DCF group. However, after adjustment for baseline imbalances by matched-pair and stratified analyses, these differences were no longer statistically significant. Therefore, the apparent advantage of FLOT should be interpreted with caution, as it may be confounded by tumor location and clinical stage.

The pCR rate in the FLOT group was 20%, comparable to the FLOT4 trial (16%) [12]. The DCF group showed a lower pCR rate (2%), which may have contributed to the shorter DFS and OS. Although the difference was not statistically significant, OS also tended to be better in the FLOT group. Only 20% of patients in the FLOT group received adjuvant chemotherapy, which could have influenced survival outcomes.

The FLOT4 and ESOPEC trials have established FLOT as a superior regimen over ECX/ECF and NACRT in EAC [12, 13]. In our cohort, clinical stage and tumor location biases were adjusted using matched-pair analysis, showing consistent results without significant impact from Siewert classification [25].

In the FLOT group, four patients received adjuvant chemotherapy, whereas no patients received adjuvant chemotherapy in the DCF group, which may have influenced the DFS. At the time when DCF therapy was used, treatment strategies were based mainly on preoperative chemotherapy similar to those for EC. In an effort to further improve outcomes, we subsequently introduced adjuvant chemotherapy based on gastric cancer regimens. During the FLOT era, adjuvant chemotherapy was implemented for non-pCR cases, although due to patients’ postoperative condition and preferences, it was not feasible for all eligible patients.

The incidence of grade ≥ 3 Adverse events (AEs) did not differ significantly between the two groups. However, among any grade AEs, nausea, anorexia, stomatitis/mucositis, and increased serum creatinine occurred significantly more frequently in the DCF group, whereas peripheral neuropathy was more frequent in the FLOT group; all of these events were manageable. Grade ≥ 3 neutropenia was similar to previous FLOT reports in ESCC (65%) [25], and febrile neutropenia (FN) was 0% in FLOT and 12.5% in DCF. For FLOT, no consensus on appropriate supportive care has been established to date; based on our single-institution experience [26], we adopted prophylactic G-CSF. Primary prophylactic G-CSF was more frequently used in the FLOT group (85% vs. 3%), which may explain the lower FN rate, and no prophylactic antibiotics were routinely used.

In the DCF group, as in JCOG1109, prophylactic antibiotics were routinely used, and we found that the incidence of FN could be controlled without routine G-CSF administration. In JCOG1109, grade ≥ 3 neutropenia and FN were 85% and 16% with DCF [22], aligning with our findings. DCF can be administered with proper management. Treatment completion rates and relative dose intensity (RDI) were comparable between groups.

Looking ahead, the perioperative landscape continues to evolve. The MATTERHORN trial recently showed that FLOT plus durvalumab significantly improved event-free survival (EFS) compared to FLOT alone (HR 0.71, p < 0.001) [27]. FLOT plus immunotherapy may become the new standard for resectable GC/EGJ-AC.

This study has limitations: its retrospective, single-center nature, small sample size, and short follow-up. The decision to provide adjuvant therapy was physician-dependent, which may influence survival analyses. Nonetheless, our findings contribute important comparative data on neoadjuvant triplet chemotherapy regimens in Japanese patients with EGJ-AC.

## Conclusion

Neoadjuvant FLOT therapy for EGJ-AC is well-tolerated and demonstrates favorable short-term efficacy and tolerability compared to neoadjuvant DCF therapy.

## Data Availability

The data that support the findings of this study are available from the corresponding author upon reasonable request.
